# Bostrycin

**DOI:** 10.1107/S1600536808032030

**Published:** 2008-10-31

**Authors:** Shao-Yuan Chen, Chao Huang, Chu-Long Zhang, Yu-Zhe Chen, Fu-Cheng Lin

**Affiliations:** a State Key Laboratory for Rice Biology, Institute of Biotechnology, Zhejiang University, People’s Republic of China; b College of Pharmaceutical Sicence, Zhejiang University, People’s Republic of China

## Abstract

The title compound, C_16_H_16_O_8_, is a potent nonspecific phyto­toxin. The crystal structure is the average of two tauto­mers, 5,6,7,9,10-penta­hydr­oxy-2-meth­oxy-7-methyl-1,4,5,6,7,8-hexa­hydro­anthracene-1,4-dione and 1,4,5,6,7-pentahydr­oxy-2-meth­­oxy-7-methyl-5,6,7,8,9,10-hexa­hydro­anthracene-9,10-di­one. The cyclo­hexene rings in both tautomers display a half-chair conformation. An extensive O—H⋯O hydrogen-bonding network is present in the crystal structure.

## Related literature

For general background, see: Charudattan & Rao (1982 ▶); van Eijk (1975 ▶). For a related structure, see: Kelly & Saha (1985 ▶).
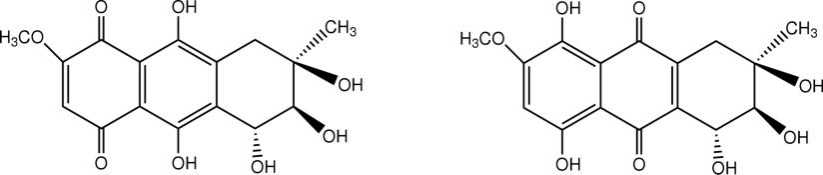


## Experimental

### 

#### Crystal data

C_16_H_16_O_8_
*M* *_r_* = 336.29
Monoclinic, 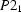

*a* = 8.280 (2) Å
*b* = 6.644 (2) Å
*c* = 13.1535 (12) Å
β = 102.12 (2)°
*V* = 707.5 (3) Å^3^
*Z* = 2
Mo *K*α radiation
μ = 0.13 mm^−1^
*T* = 293 (2) K
0.30 × 0.20 × 0.08 mm

#### Data collection

Rigaku R-AXIS RAPID IP diffractometer
Absorption correction: multi-scan (*ABSCOR*; Higashi, 1995 ▶) *T*_min_ = 0.953, *T*_max_ = 0.990
6151 measured reflections
1517 independent reflections
1282 reflections with *I* > 2σ(*I*)
*R*_int_ = 0.025

#### Refinement

*R*[*F* ^2^ > 2σ(*F* ^2^)] = 0.035
*wR*(*F* ^2^) = 0.105
*S* = 1.08
1517 reflections
220 parameters
1 restraint
H-atom parameters constrained
Δρ_max_ = 0.24 e Å^−3^
Δρ_min_ = −0.19 e Å^−3^

### 

Data collection: *PROCESS-AUTO* (Rigaku, 1998 ▶); cell refinement: *PROCESS-AUTO*; data reduction: *CrystalStructure* (Rigaku/MSC, 2002 ▶); program(s) used to solve structure: *SHELXS97* (Sheldrick, 2008 ▶); program(s) used to refine structure: *SHELXL97* (Sheldrick, 2008 ▶); molecular graphics: *ORTEP-3* (Farrugia, 1997 ▶); software used to prepare material for publication: *WinGX* (Farrugia, 1999 ▶).

## Supplementary Material

Crystal structure: contains datablocks I, global. DOI: 10.1107/S1600536808032030/xu2453sup1.cif

Structure factors: contains datablocks I. DOI: 10.1107/S1600536808032030/xu2453Isup2.hkl

Additional supplementary materials:  crystallographic information; 3D view; checkCIF report

## Figures and Tables

**Table 1:** Hydrogen-bond geometry (Å, °)

*D*—H⋯*A*	*D*—H	H⋯*A*	*D*⋯*A*	*D*—H⋯*A*
O1—H1*A*⋯O3^i^	0.85	2.55	3.229 (3)	137
O3—H3*A*⋯O8^ii^	0.84	2.40	2.808 (3)	111
O4—H4*A*⋯O8^ii^	0.90	2.54	3.223 (3)	132
O5—H5*A*⋯O4	0.92	1.85	2.687 (3)	152
O6—H6*A*⋯O7^iii^	0.97	1.92	2.821 (3)	154
O7—H7*A*⋯O1^iv^	0.90	2.11	2.966 (3)	159
O7—H7*A*⋯O2^iv^	0.90	2.40	3.066 (3)	131

Symmetry codes: (i) 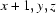; (ii) 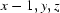; (iii) 
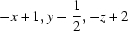
; (iv) 
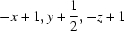
.

**Fig. 1.**
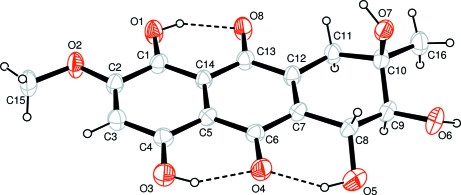
The molecular structure of (I) with 50% probability displacement ellipsoids (arbitrary spheres for H atoms), dashed line indicates hydrogen bonding.

**Fig. 2.**
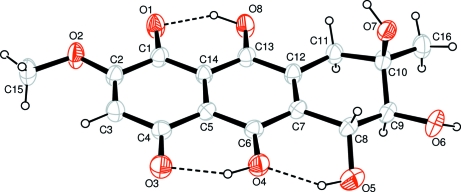
The molecular structure of (II) with 50% probability displacement ellipsoids (arbitrary spheres for H atoms), dashed line indicates hydrogen bonding.

## supplementary crystallographic information

### Comment

Bostrycin, a nonspecific phytotoxin, was identified as a metabolite of the fungus Arthrinium phaeospermum in 1975 (van Eijk,  1975; Charudattan & Rao, 1982). It is active against Bacillus subtilis but inactive against the fungus Geotrichum candidum (Charudattan & Rao, 1982). We report here the crystal structure of the title compound.

The crystal structure of the title compound is the average structure of two tautomers, 5,6,7,9,10-pentahydroxy-2-methoxy-7-methyl-1,4,5,6,7,8-hexahydroanthracene- 1,4-dione (I) and 1,4,5,6,7-pentahydroxy-2-methoxy-7-methyl-5,6,7,8,9,10-hexahydroanthracene- 9,10-dione (II). The molecular structures of the two tautomers are shown in Fig. 1 and Fig. 2, respectively. Both of tautomer molecules contains three six-membered rings, among which the C9-containing ring displays a half-chair conformation. Within the molecule the carbonyl group is hydrogen bonded to the neighboring hydroxyl group(s). The bond distances and angles agree with those found in a derivative of bostrycin, bostrycin acetonide (Kelly & Saha, 1985). The extensive O—H···O hydrogen bonding network helps to stabilize the crystal structure (Table 1).

### Experimental

For morphological identification (Arthrinium *sp*. (CGMCC 2082), a fungi from Polygonum hydropiper *L*.) cultures were grown on OA, PDA, and SNA media for 7–14 days at room temperature (293 K) under ambient daylight. Microscopic observations and measurements were made from slides mounted in water. For metabolite production, the strains were inoculated onto PDA media and incubated for 10 days at 298 K in the dark. Selected strains were also cultivated in liquid media placed in a rotary shaker at 120 rpm for 7 days at 298 K in the dark. After cultivation, the bottles were stored at 253 K until extraction.

Liquid cultures were extracted with trichloromethane. The trichloromethane phase was filtered and evaporated *in vacuo*. Samples were then redissolved in trichloromethane, then filtered to remove solid. The trichloromethane solution was evaporated *in vacuo*. The single crystals were obtained from an ethanol solution.

### Refinement

Hydroxyl H atoms were located in a difference Fourier map and refined as riding in as-found relative positions with *U*_iso_(H) = 1.5*U*_eq_(O). Methyl H atoms were placed in calculated positions with C—H = 0.96 Å and torsion angles were refined to fit the electron density, *U*_iso_(H) = 1.5*U*_eq_(C). Other H atoms were placed in calculated positions with C—H = 0.93 (aromatic), 0.97 (methylene) or 0.98 Å (methine), and refined in riding mode with *U*_iso_(H) = 1.2*U*_eq_(C). In the absence of significant anomalous scattering effects, Friedel pairs were merged; the absolute configuration was not determined.

### Crystal data

**Table:**

C_16_H_16_O_8_	*F*(000) = 352
*M**_r_* = 336.29	*D*_x_ = 1.579 Mg m^−^^3^
Monoclinic, *P*2_1_	Mo *K*α radiation, λ = 0.71069 Å
Hall symbol: P 2yb	Cell parameters from 5814 reflections
*a* = 8.280 (2) Å	θ = 6.1–54.9°
*b* = 6.644 (2) Å	µ = 0.13 mm^−^^1^
*c* = 13.1535 (12) Å	*T* = 293 K
β = 102.12 (2)°	Chunk, red
*V* = 707.5 (3) Å^3^	0.30 × 0.20 × 0.08 mm
*Z* = 2	

### Data collection

**Table:**

Rigaku R-AXIS RAPID IP diffractometer	1517 independent reflections
Radiation source: fine-focus sealed tube	1282 reflections with *I* > 2σ(*I*)
graphite	*R*_int_ = 0.025
Detector resolution: 10 pixels mm^-1^	θ_max_ = 26.0°, θ_min_ = 3.2°
ω scans	*h* = −10→10
Absorption correction: multi-scan (ABSCOR; Higashi, 1995)	*k* = −8→8
*T*_min_ = 0.953, *T*_max_ = 0.990	*l* = −16→16
6151 measured reflections	

### Refinement

**Table:**

Refinement on *F*^2^	Secondary atom site location: difference Fourier map
Least-squares matrix: full	Hydrogen site location: inferred from neighbouring sites
*R*[*F*^2^ > 2σ(*F*^2^)] = 0.035	H-atom parameters constrained
*wR*(*F*^2^) = 0.105	*w* = 1/[σ^2^(*F*_o_^2^) + (0.0667*P*)^2^ + 0.0817*P*] where *P* = (*F*_o_^2^ + 2*F*_c_^2^)/3
*S* = 1.08	(Δ/σ)_max_ = 0.001
1517 reflections	Δρ_max_ = 0.24 e Å^−^^3^
220 parameters	Δρ_min_ = −0.19 e Å^−^^3^
1 restraint	Extinction correction: SHELXL97 (Sheldrick, 2008), Fc^*^=kFc[1+0.001xFc^2^λ^3^/sin(2θ)]^-1/4^
Primary atom site location: structure-invariant direct methods	Extinction coefficient: 0.009 (3)

### Special details

**Table:**

Geometry. All e.s.d.'s (except the e.s.d. in the dihedral angle between two l.s. planes) are estimated using the full covariance matrix. The cell e.s.d.'s are taken into account individually in the estimation of e.s.d.'s in distances, angles and torsion angles; correlations between e.s.d.'s in cell parameters are only used when they are defined by crystal symmetry. An approximate (isotropic) treatment of cell e.s.d.'s is used for estimating e.s.d.'s involving l.s. planes.
Refinement. Refinement of *F*^2^ against ALL reflections. The weighted *R*-factor *wR* and goodness of fit *S* are based on *F*^2^, conventional *R*-factors *R* are based on *F*, with *F* set to zero for negative *F*^2^. The threshold expression of *F*^2^ > σ(*F*^2^) is used only for calculating *R*-factors(gt) *etc*. and is not relevant to the choice of reflections for refinement. *R*-factors based on *F*^2^ are statistically about twice as large as those based on *F*, and *R*- factors based on ALL data will be even larger.

### Fractional atomic coordinates and isotropic or equivalent isotropic displacement parameters (Å^2^)

**Table:**

	*x*	*y*	*z*	*U*_iso_*/*U*_eq_	Occ. (<1)
O1	0.4354 (2)	0.3424 (4)	0.34450 (13)	0.0399 (5)	
H1A	0.5153	0.3341	0.3968	0.060*	0.50
O2	0.1814 (2)	0.3555 (4)	0.19361 (12)	0.0414 (5)	
O3	−0.1725 (2)	0.3863 (4)	0.43528 (13)	0.0384 (5)	
H3A	−0.1659	0.3844	0.4995	0.058*	0.50
O4	−0.0420 (2)	0.3933 (4)	0.63061 (13)	0.0370 (5)	
H4A	−0.1226	0.4012	0.5725	0.055*	0.50
O5	0.1000 (3)	0.2810 (5)	0.82507 (17)	0.0571 (7)	
H5A	0.0233	0.2963	0.7644	0.086*	
O6	0.3619 (2)	0.4004 (4)	0.97960 (12)	0.0417 (5)	
H6A	0.4364	0.3205	1.0303	0.063*	
O7	0.5068 (2)	0.6526 (3)	0.84879 (13)	0.0336 (5)	
H7A	0.5511	0.7105	0.7990	0.050*	
O8	0.5671 (2)	0.3429 (4)	0.54013 (13)	0.0401 (6)	
H8A	0.5559	0.3636	0.4666	0.060*	0.50
C1	0.2943 (3)	0.3552 (4)	0.37142 (18)	0.0302 (6)	
C2	0.1464 (3)	0.3632 (4)	0.28875 (18)	0.0303 (6)	
C3	−0.0063 (3)	0.3742 (5)	0.31216 (18)	0.0314 (6)	
H3	−0.0996	0.3781	0.2587	0.038*	
C4	−0.0247 (3)	0.3798 (4)	0.41735 (18)	0.0284 (5)	
C5	0.1177 (3)	0.3742 (4)	0.50050 (17)	0.0263 (5)	
C6	0.1023 (3)	0.3820 (4)	0.60602 (17)	0.0268 (5)	
C7	0.2492 (3)	0.3761 (5)	0.68900 (17)	0.0291 (6)	
C8	0.2301 (3)	0.3980 (5)	0.80142 (17)	0.0311 (6)	
H8	0.2063	0.5396	0.8130	0.037*	
C9	0.3863 (3)	0.3403 (4)	0.87974 (18)	0.0323 (6)	
H9	0.3996	0.1938	0.8790	0.039*	
C10	0.5382 (3)	0.4387 (4)	0.85300 (17)	0.0309 (6)	
C11	0.5585 (3)	0.3595 (5)	0.74721 (17)	0.0319 (6)	
H11A	0.6425	0.4383	0.7240	0.038*	
H11B	0.5970	0.2213	0.7552	0.038*	
C12	0.4022 (3)	0.3671 (4)	0.66537 (17)	0.0294 (6)	
C13	0.4195 (3)	0.3563 (4)	0.55826 (18)	0.0296 (6)	
C14	0.2770 (3)	0.3626 (4)	0.47627 (17)	0.0262 (5)	
C15	0.0456 (3)	0.3549 (6)	0.10420 (18)	0.0435 (8)	
H15A	−0.0220	0.2384	0.1068	0.065*	
H15B	0.0881	0.3517	0.0417	0.065*	
H15C	−0.0196	0.4743	0.1048	0.065*	
C16	0.6936 (3)	0.3933 (6)	0.93488 (19)	0.0408 (7)	
H16A	0.7885	0.4434	0.9117	0.061*	
H16B	0.7039	0.2505	0.9453	0.061*	
H16C	0.6861	0.4575	0.9991	0.061*	

### Atomic displacement parameters (Å^2^)

**Table:**

	*U*^11^	*U*^22^	*U*^33^	*U*^12^	*U*^13^	*U*^23^
O1	0.0294 (10)	0.0644 (15)	0.0268 (8)	0.0039 (10)	0.0076 (7)	−0.0058 (10)
O2	0.0318 (10)	0.0719 (15)	0.0203 (7)	0.0052 (11)	0.0050 (7)	−0.0025 (10)
O3	0.0233 (9)	0.0584 (13)	0.0343 (9)	0.0031 (10)	0.0082 (7)	0.0005 (10)
O4	0.0279 (10)	0.0541 (12)	0.0311 (8)	−0.0002 (10)	0.0112 (7)	−0.0016 (10)
O5	0.0513 (14)	0.0811 (19)	0.0421 (11)	−0.0192 (13)	0.0170 (10)	0.0069 (12)
O6	0.0528 (12)	0.0518 (12)	0.0226 (8)	0.0093 (11)	0.0126 (8)	0.0056 (10)
O7	0.0444 (11)	0.0318 (10)	0.0262 (8)	−0.0028 (9)	0.0112 (7)	0.0001 (8)
O8	0.0223 (9)	0.0710 (16)	0.0279 (8)	0.0015 (10)	0.0073 (7)	−0.0065 (11)
C1	0.0294 (13)	0.0370 (15)	0.0262 (11)	0.0026 (13)	0.0101 (9)	−0.0003 (12)
C2	0.0326 (14)	0.0360 (15)	0.0233 (11)	0.0014 (14)	0.0079 (9)	−0.0003 (12)
C3	0.0298 (13)	0.0360 (14)	0.0274 (11)	0.0032 (13)	0.0035 (9)	0.0003 (12)
C4	0.0249 (13)	0.0297 (13)	0.0307 (11)	0.0012 (12)	0.0064 (9)	0.0003 (12)
C5	0.0274 (13)	0.0282 (12)	0.0249 (11)	0.0006 (12)	0.0089 (9)	0.0005 (12)
C6	0.0267 (12)	0.0279 (12)	0.0278 (11)	−0.0006 (12)	0.0103 (9)	−0.0021 (12)
C7	0.0317 (13)	0.0334 (13)	0.0245 (11)	−0.0020 (13)	0.0108 (9)	−0.0003 (12)
C8	0.0335 (14)	0.0369 (14)	0.0261 (11)	−0.0011 (12)	0.0137 (10)	0.0017 (12)
C9	0.0411 (15)	0.0348 (14)	0.0232 (10)	0.0016 (13)	0.0117 (10)	0.0021 (11)
C10	0.0363 (15)	0.0338 (15)	0.0224 (12)	0.0012 (12)	0.0055 (10)	0.0004 (11)
C11	0.0283 (13)	0.0429 (16)	0.0251 (11)	0.0016 (13)	0.0067 (9)	−0.0028 (12)
C12	0.0304 (13)	0.0340 (14)	0.0248 (11)	0.0024 (13)	0.0079 (9)	−0.0028 (12)
C13	0.0270 (13)	0.0370 (15)	0.0262 (11)	0.0028 (12)	0.0087 (9)	−0.0019 (11)
C14	0.0250 (12)	0.0309 (13)	0.0230 (10)	0.0014 (12)	0.0059 (9)	−0.0019 (12)
C15	0.0381 (15)	0.068 (2)	0.0225 (11)	0.0052 (17)	0.0028 (10)	−0.0018 (15)
C16	0.0368 (15)	0.0555 (17)	0.0279 (12)	0.0023 (15)	0.0019 (10)	−0.0011 (14)

### Geometric parameters (Å, °)

**Table:**

O1—C1	1.293 (3)	C5—C6	1.421 (3)
O1—H1A	0.8501	C5—C14	1.423 (3)
O2—C2	1.344 (3)	C6—C7	1.454 (3)
O2—C15	1.447 (3)	C7—C12	1.368 (4)
O3—C4	1.294 (3)	C7—C8	1.527 (3)
O3—H3A	0.8350	C8—C9	1.524 (4)
O4—C6	1.304 (3)	C8—H8	0.9800
O4—H4A	0.9046	C9—C10	1.523 (4)
O5—C8	1.415 (4)	C9—H9	0.9800
O5—H5A	0.9154	C10—C16	1.525 (3)
O6—C9	1.427 (3)	C10—C11	1.530 (3)
O6—H6A	0.9667	C11—C12	1.501 (3)
O7—C10	1.444 (3)	C11—H11A	0.9700
O7—H7A	0.9012	C11—H11B	0.9700
O8—C13	1.296 (3)	C12—C13	1.448 (3)
O8—H8A	0.9624	C13—C14	1.422 (3)
C1—C14	1.417 (3)	C15—H15A	0.9600
C1—C2	1.458 (3)	C15—H15B	0.9600
C2—C3	1.364 (4)	C15—H15C	0.9600
C3—C4	1.424 (3)	C16—H16A	0.9600
C3—H3	0.9300	C16—H16B	0.9600
C4—C5	1.431 (3)	C16—H16C	0.9600
			
C1—O1—H1A	112.1	C10—C9—C8	111.2 (2)
C2—O2—C15	118.3 (2)	O6—C9—H9	108.9
C4—O3—H3A	108.7	C10—C9—H9	108.9
C6—O4—H4A	110.2	C8—C9—H9	108.9
C8—O5—H5A	99.7	O7—C10—C9	106.3 (2)
C9—O6—H6A	106.8	O7—C10—C16	109.9 (2)
C10—O7—H7A	110.5	C9—C10—C16	111.6 (2)
C13—O8—H8A	106.3	O7—C10—C11	110.9 (2)
O1—C1—C14	123.4 (2)	C9—C10—C11	108.4 (2)
O1—C1—C2	117.6 (2)	C16—C10—C11	109.7 (2)
C14—C1—C2	119.0 (2)	C12—C11—C10	113.5 (2)
O2—C2—C3	127.1 (2)	C12—C11—H11A	108.9
O2—C2—C1	112.4 (2)	C10—C11—H11A	108.9
C3—C2—C1	120.4 (2)	C12—C11—H11B	108.9
C2—C3—C4	120.9 (2)	C10—C11—H11B	108.9
C2—C3—H3	119.5	H11A—C11—H11B	107.7
C4—C3—H3	119.5	C7—C12—C13	120.6 (2)
O3—C4—C3	118.4 (2)	C7—C12—C11	122.6 (2)
O3—C4—C5	121.4 (2)	C13—C12—C11	116.7 (2)
C3—C4—C5	120.2 (2)	O8—C13—C14	121.8 (2)
C6—C5—C14	119.9 (2)	O8—C13—C12	118.2 (2)
C6—C5—C4	121.1 (2)	C14—C13—C12	120.1 (2)
C14—C5—C4	119.0 (2)	C1—C14—C13	120.0 (2)
O4—C6—C5	121.3 (2)	C1—C14—C5	120.5 (2)
O4—C6—C7	118.7 (2)	C13—C14—C5	119.5 (2)
C5—C6—C7	120.0 (2)	O2—C15—H15A	109.5
C12—C7—C6	119.9 (2)	O2—C15—H15B	109.5
C12—C7—C8	120.9 (2)	H15A—C15—H15B	109.5
C6—C7—C8	119.0 (2)	O2—C15—H15C	109.5
O5—C8—C9	106.9 (2)	H15A—C15—H15C	109.5
O5—C8—C7	113.5 (2)	H15B—C15—H15C	109.5
C9—C8—C7	112.6 (2)	C10—C16—H16A	109.5
O5—C8—H8	107.8	C10—C16—H16B	109.5
C9—C8—H8	107.8	H16A—C16—H16B	109.5
C7—C8—H8	107.8	C10—C16—H16C	109.5
O6—C9—C10	112.1 (2)	H16A—C16—H16C	109.5
O6—C9—C8	106.7 (2)	H16B—C16—H16C	109.5

### Hydrogen-bond geometry (Å, °)

**Table:**

*D*—H···*A*	*D*—H	H···*A*	*D*···*A*	*D*—H···*A*
O1—H1A···O3^i^	0.85	2.55	3.229 (3)	137
O3—H3A···O8^ii^	0.84	2.40	2.808 (3)	111
O4—H4A···O8^ii^	0.90	2.54	3.223 (3)	132
O5—H5A···O4	0.92	1.85	2.687 (3)	152
O6—H6A···O7^iii^	0.97	1.92	2.821 (3)	154
O7—H7A···O1^iv^	0.90	2.11	2.966 (3)	159
O7—H7A···O2^iv^	0.90	2.40	3.066 (3)	131

Symmetry codes: (i) *x*+1, *y*, *z*; (ii) *x*−1, *y*, *z*; (iii) −*x*+1, *y*−1/2, −*z*+2; (iv) −*x*+1, *y*+1/2, −*z*+1.
